# Novel automated vessel pattern characterization of larynx contact endoscopic video images

**DOI:** 10.1007/s11548-019-02034-9

**Published:** 2019-07-27

**Authors:** Nazila Esmaeili, Alfredo Illanes, Axel Boese, Nikolaos Davaris, Christoph Arens, Michael Friebe

**Affiliations:** 1 INKA, Institute of Medical Technology, Otto-von-Guericke University Magdeburg, Magdeburg, Germany; 2 Department of Otorhinolaryngology, Head and Neck Surgery, Magdeburg University Hospital, Magdeburg, Germany

**Keywords:** Contact endoscopy, Larynx, Vascular pattern, Feature extraction, Classification

## Abstract

**Purpose:**

Contact endoscopy (CE) is a minimally invasive procedure providing real-time information about the cellular and vascular structure of the superficial layer of laryngeal mucosa. This method can be combined with optical enhancement methods such as narrow band imaging (NBI). However, these techniques have some problems like subjective interpretation of vascular patterns and difficulty in differentiation between benign and malignant lesions. We propose a novel automated approach for vessel pattern characterization of larynx CE + NBI images in order to solve these problems.

**Methods:**

In this approach, five indicators were computed to characterize the level of vessel’s disorder based on evaluation of consistency of gradient and two-dimensional curvature analysis and then 24 features were extracted from these indicators. The method evaluated the ability of the extracted features to classify CE + NBI images based on the vascular pattern and based on the laryngeal lesions. Four datasets were generated from 32 patients involving 1485 images. The classification scenarios were implemented using four supervised classifiers.

**Results:**

For classification of CE + NBI images based on the vascular pattern, polykernel support vector machine (SVM), SVM with radial basis function (RBF), k-nearest neighbor (kNN), and random forest (RF) show an accuracy of 97%, 96%, 96%, and 96%, respectively. For the classification based on the histopathology, Polykernel SVM showed an accuracy of 84%, 86% and 84%, RBF SVM showed an accuracy of 81%, 87% and 83%, kNN showed an accuracy of 89%, 87%, 91%, RF showed an accuracy of 90%, 88% and 91% for classification between benign histopathologies, between malignant histopathologies and between benign and malignant lesions, respectively.

**Conclusion:**

These promising results show that the proposed method could solve the problem of subjectivity in interpretation of vascular patterns and also support the clinicians in the early detection of benign, pre-malignant and malignant lesions.

## Introduction

The larynx (voice box) is part of the head and neck region, and laryngeal cancer belongs to the most common cancer types with high incidence and mortality. Precancerous lesions such as laryngeal dysplasia precede the development of laryngeal cancer. 85–95% of laryngeal cancers are squamous cell carcinomas (SCC) [1]. Early detection and diagnosis of suspicious mucosal lesions could provide an important opportunity to preserve the larynx and vocal fold function. Histopathological examination of suspicious laryngeal tissue using surgical biopsy is currently the gold standard for diagnosis, which is an invasive procedure and can cause serious problems for the patient [2].

The development of larynx endoscopy techniques provide a minimally invasive examination along with the possibility of early detection of vocal fold disorders. Barbalata and Mattos [3] proposed a method for laryngeal tumor detection and classification in narrow band imaging (NBI) endoscopic images. They reported an accuracy of 84.3% in recognizing malignant laryngeal tumors based on vascular characterization of the tumor. Turkmen et al. [4] proposed an approach to classify vocal fold disorders based on visible blood vessels and shape of vocal fold edges, with a sensitivity of 86%, 94%, 80%, 73%, and 76% for healthy, polyp, nodule, laryngitis, and sulcus vocalis classes, respectively. Moccia et al. [5] applied texture-based and first-order statistical features on $100\times 100$ px patches in NBI endoscopic images to classify laryngeal tissue into four classes: tissue with intraepithelial papillary capillary loop (IPCL)-like vessels, leukoplakia, tissue with hypertrophic vessels and healthy tissue. They used support vector machine (SVM) classifier and reported achieved median classification recall of 93% with the best performing feature. In a recent study by Nanni et al. [6], an ensemble of convolutional neural networks (CNNs) and handcrafted features for bioimage classification was proposed. This ensemble obtained promising performance on the NBI endoscopic images dataset [5] with 97.33% accuracy to differentiate between four laryngeal tissue classes. Despite all the advantages, standalone application of normal white light video laryngoscopy or NBI cannot provide highly magnified visualization of color, contour, texture, and extent of mucosal lesions. For this reason, there is a need to have a technique that provides more precise evaluation of histopathology of laryngeal tissue for differential diagnosis of laryngeal cancerous lesions.

Contact endoscopy (CE) is an optical technique that allows detailed examination of the superficial layers of laryngeal mucosa providing a visualization of cells and vascular structures. This procedure is regularly performed using white light imaging, but it can also be combined with optical enhancement technologies like NBI. NBI is able to increase tissue contrast and to enhance the superficial vascular pattern at the site of examination [7]. The first application of CE in the larynx was reported in [8] and its efficiency was subsequently confirmed as a diagnostic tool in the evaluation of various pathologies in the larynx [9].

**Fig. 1:**
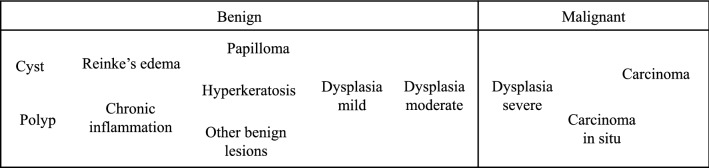
A classification for laryngeal histopathology used at the University Hospital Magdeburg. The severity increases from left to right

**Fig. 2:**
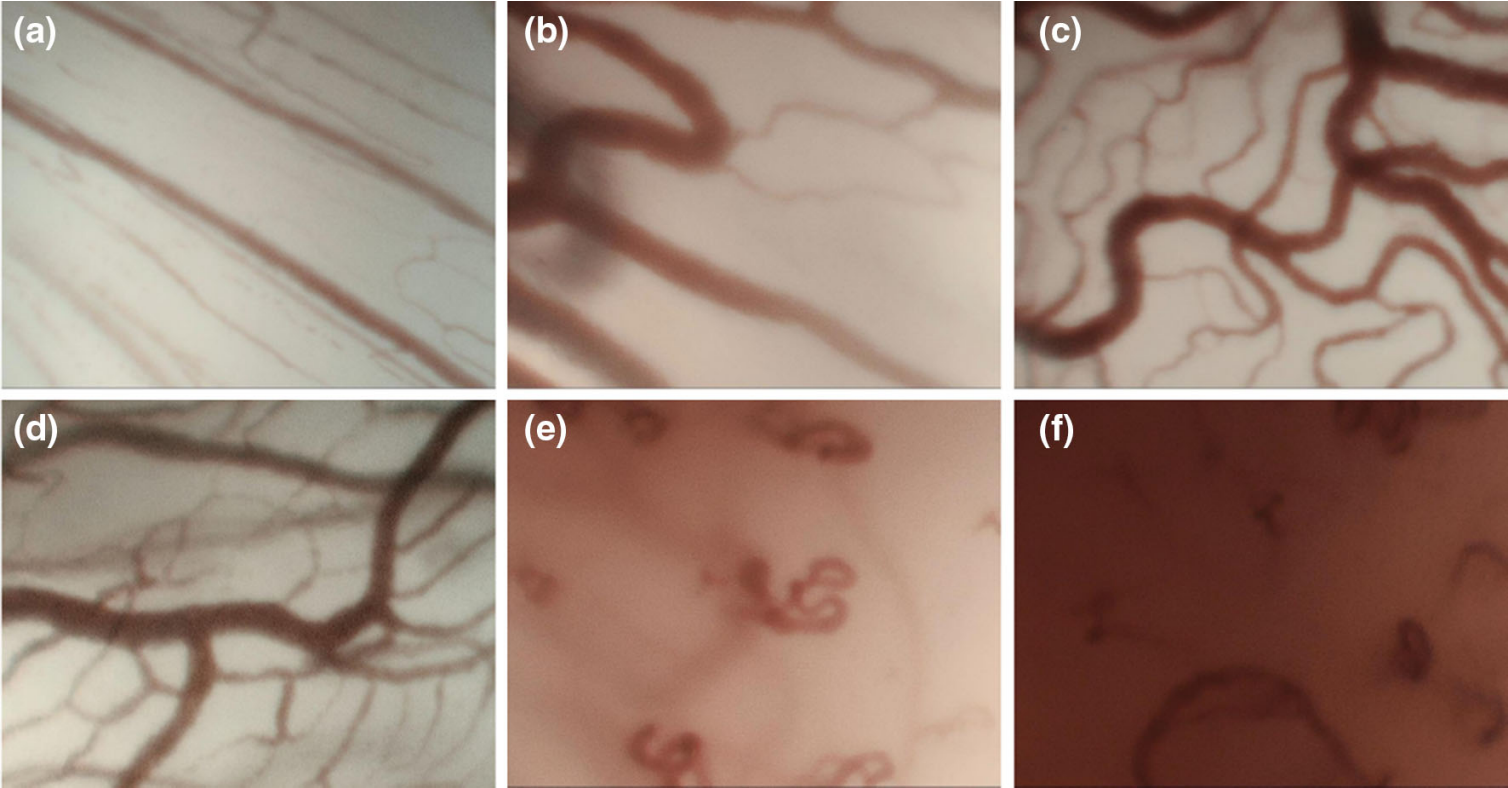
Examples of CE + NBI images of six different cases: **a** healthy, **b** polyp, **c** reinke’s edema, **d** dysplasia mild, **e** carcinoma in situ, **f** carcinoma

In the early application of larynx CE, the main focus was on finding histopathological information by evaluating the cellular architecture of the tissue. An example of that is the study [10], where a computer-assisted image analysis for diagnosis of precancerous and cancerous lesions based on the characterization of the cellular architecture in CE images was used. Recent studies showed that the evaluation of vascular patterns of the larynx superficial network can provide the surgeons more information than the cellular field. This is because the structure and the organization of blood vessels in the vocal fold is dynamic and undergoes significant changes in non-cancerous and cancerous stages [11, 12]. Puxeddu et al. [13] visually classified vascular patterns in enhanced contact endoscopy (ECE) images into five categories for differential diagnosis between normal tissue and hyperplasia versus mild dysplasia and carcinoma. But, there is no study on automatic classification of the CE vascular patterns.

It is recognized that the limiting factors of CE prevented it to gain acceptable place in routine clinical practice despite its potential advantages. Interpretation and evaluation of CE require extensive learning from the clinicians [14, 15]. Studies showed that at the beginning of the training, there is a risk of subjective interpretation of vascular patterns [13, 16]. This problem may cause an increased number of false positives which results in unnecessary biopsies [17]. Also, difficulty in differentiation between hyperplasia and mild-to-moderate dysplasia as well as an inability in differentiation of carcinoma in situ from carcinoma was reported [2, 13, 15].

The main objective of this work is to automatically characterize and assess vascular patterns in CE + NBI images to classify images based on the vascular pattern and laryngeal histopathology. For this, a new algorithm is proposed to evaluate the level of disorder in vessels based on the consistency of gradient direction and the vessels’ curvature. Five indicators were computed after image preprocessing and vessel segmentation and then 24 features were extracted based on the qualitative properties of the indicators. The extracted features were fed into four different classifiers to classify images based on the vascular pattern and on larynx histopathology.

## Material and methods

### Data acquisition

Video scenes of 32 patients presenting different primary diagnosis were acquired during the examination of vocal folds with a frame rate equal to 30 frame per seconds (fps) in the department of Otorhinolaryngology at the University Hospital Magdeburg. A contact endoscope (KARL STORZ, Tuttlingen, Germany) in combination with an endoscopic imaging system (VISERA 4K UHD, Olympus, Japan) was used to capture the video scenes in Audio Video Interleave (AVI) format. In all procedures, the magnification of the contact endoscope was fixed at $60\times$ in order to have a fixed camera–tissue distance. For each patient, video segments where contact endoscope was used were manually extracted. Inside the video segments, we manually selected the intervals where the video quality was acceptable to see the vessels (good resolution without blur and artifacts). Then, one frame every three frames were extracted from the selected intervals in JPEG format images ($1008 \times 1280$ px) and stored in the patient datasets to use them for the further processing.

Patients’ data were pseudonymized, and only biopsy results were used as the final diagnosis for each patient, based on the classification of laryngeal histopathologies used by the medical doctors at the Magdeburg University Hospital (Fig. 1). In this classification, laryngeal histopathologies are divided into two main groups: benign and malignant, which each of them subdivided in different histopathologies.

### Image preprocessing and indicators extraction

**Fig. 3:**
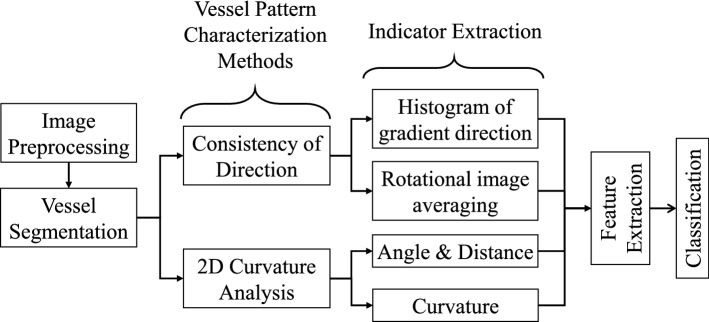
Block diagram with the main steps of the proposed approach

Figure 2 shows examples of vocal fold images extracted from videos belonging to 6 different histopathologies. One of the main characteristics that clinicians observe in these images is the level of disorder in vessels. In conversation with our clinicians and based on recent publications, where vessel patterns were manually analyzed and classified [12–14], 5 indicators were proposed for characterizing vessel patterns. These indicators intended to take into account geometrical characteristics to assess the level of disorder of vascular patterns. The main idea was to extract features from the indicators and use them for classifying images according to the vessel’s level of disorder and laryngeal histopathology. Figure 3 shows the main steps for the automatic feature extraction and classification procedures which are subsequently described in more details.

**Fig. 4:**
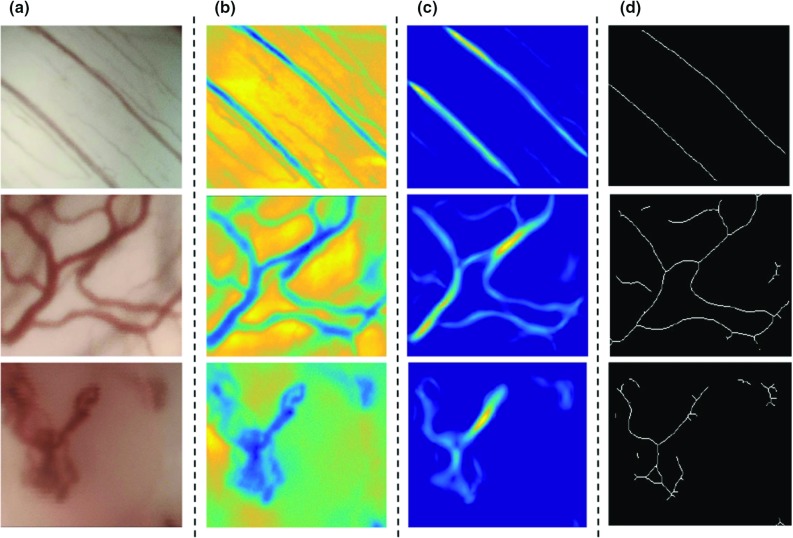
Image preprocessing for three different vascular patterns in CE + NBI images: **a** original image, **b** homogenization, **c** Frangi filter, **d** skeletonization

#### Image preprocessing and vessel segmentation

In order to remove the very low frequency trend in the image, a Daubechies level 7 discrete wavelet transformation was first applied to detrend each row and column of the image matrix [18]. Then a Frangi filter was used for vessel enhancement [19]. Frangi filter is a multiscale method using second order local structure of an image (Hessian) to find tubular structures as well as first-order transaction (gradient vector) to estimate the direction of these structures. In the image, vessels appear in different sizes. So it is important to have a measurement scale (Sigma) which varies within certain range in order to cover all different width and detect all vessels. The empirical tests performed in [19] showed that the range of Sigma between 1 to 8 can cover all the possible vascular structures. In this study, we have set the Sigma to the already tested values in order to extract the vessels in CE + NBI images. The resulting image was converted to a binary image followed by a skeletonization procedure using iterative thinning to reduce vessels to one-pixel-wide lines. This step resulted in three processed images: enhanced, filtered, and binary skeletonized referred as $I_H$, $I_F$ and $I_S$, respectively (see examples in Fig. 4).

#### Image indicator extraction

As previously explained, five different indicators were computed to distinguish among the different vascular patterns based on direction-based and curvature-based characteristics.

*Direction-based indicators* Two indicators were based on the consistency of the vessel direction, corresponding to histogram of gradient direction (HGD) and rotational image averaging (RIA). HGD was computed over the image $I_H$ and RIA was computed over the image $I_F$.

The gradient of an image is a directional change in intensity. The image gradients are useful because the direction of gradients are more consistent (similar directions) in straight objects than in curved objects. For the HGD computation an algorithm was designed to compute magnitude and direction of the image gradients based on the method explained in [20]. The magnitude is computed to localize regions of significant gradient and the direction is used to compute an histogram of distribution of angles. The gradient direction was normalized based on the gradient magnitude values. In summary, the HGD indicator correspond to the normalized histogram of significant gradient directions.

RIA consists of computing the average over the rows of $I_F$ for different rotation angles of the image. This average should be peaky when vessels are more or less parallel at a given angle and should show flatter behavior when the vessels are more curved. For that, the image was rotated from 0 to 360 degree in steps of 45 degrees, and at each rotation the average over the image was calculated as:

1 $$\begin{aligned} s_\mathrm{row}^{\theta }(x) = \frac{1}{N}\times \sum _{y=1}^{N} I_F(x,y) \end{aligned}$$

where $s_\mathrm{row}^{\theta }$ is the resulting average row vector for the rotation angle $\theta$, $I_F(x,y)$ represents the intensity value of the pixel at the location $(x,y)$ and $N$ is the number of rows of the image. The final RIA indicator correspond to the concatenation of each $s_\mathrm{row}^{\theta }$.

*Curvature-based indicators* For these indicators, vessel segments greater than 20 px in the image $I_S$ were taken into account.

**Fig. 5:**
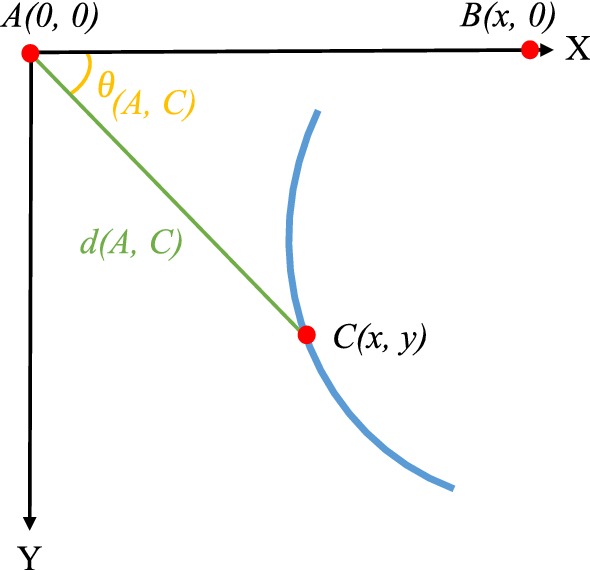
Computation of indicators ANG and DIS

The first two indicators, angle (ANG) and distance (DIS), were computed from the distance and the angle between a defined reference point (*A* in Fig. 5) and each pixel belonging to the vessel’s skeleton (*C*(*x*, *y*) in Fig. 5). The distance is simply calculated as the Euclidean distance between the reference and the skeleton point. For the angle computation a second reference point (*B* in Fig. 5) was defined and then the angle was computed between the vectors formed by the two reference and by the original reference with the skeleton point:

2 $$\begin{aligned} d(A,C)= & {} \sqrt{(x_A - x_C)^2 + (y_A - y_C)^2} \end{aligned}$$

3 $$\begin{aligned} \theta (A,C)= & {} \arctan \left( \frac{ \Vert \mathbf {\overrightarrow{AB}\times \overrightarrow{AC}} \Vert }{\overrightarrow{AB}\cdot \overrightarrow{AC}}\right) \end{aligned}$$

ANG and DIS correspond to vectors containing the resulting *d* and $\theta$ respectively for each pixel of a vessel segment. For each image an ANG and DIS vector per vessel segment is stored into a cell format.

The third curvature-based indicator, curvature (CUR), was extracted directly from the level of curvature of the vessels. For each identified segment, the curvature at each pixel point is estimated using the method presented in [21], where a global approximation of tangents using a two linear digital straight segment is applied. The CUR indicator corresponded to the concatenation of the resulting curvatures of each identified vessel segment.

## Results

### Dataset generation

Four different datasets were generated in order to evaluate the performance of the proposed approach. The approach was first validated in terms of classifying CE + NBI images based on the vascular patterns. The reason of performing this test was to evaluate the ability of the algorithm to solve the problem of subjective interpretation of vascular patterns. Then the approach was validated in terms of its suitability to classify images based on the histopathologies of the larynx, with respect to level of disorder of vessels. These tests were performed to evaluate the ability of the algorithm to solve the problems related to difficulty in differentiation between benign and malignant lesions.

**Table 1:** Histopathologies used for the generation of the three datasets

Type of cancer	Histopathology	Patients	Images	Total
Benign	Cyst	3	150	20 patients 890 images
	Polyp	4	130	
	Reinke’s edema	5	250	
	Papilloma	5	230	
	Dysplasia mild	3	130	
Malignant	Dysplasia severe	4	130	11 patients 465 images
	Carcinoma in situ	4	155	
	Carcinoma	3	180	
Total		31	1355	–

#### Dataset based on the degree of disorder of vascular patterns

*Dataset I* was generated to evaluate the performance of the proposed approach to differentiate between different degrees of disorder of vascular patterns. It included 1485 CE + NBI images from 32 patients and two medical experts came to a consensus to label them into three groups based on the vascular patterns: “order”, “disorder” and “very disorder”. “Order” vascular patterns relate to thin and parallel vessels. “Disorder” vascular patterns refer to longitudinal vascular changes. “Very disorder” vascular patterns involve perpendicular vascular pattern representing dilated IPCLs [12].

#### Dataset based on the histopathologies of the larynx

Three datasets were generated following the classification of laryngeal histopathologies (Fig. 1). Table 1 shows the different histopathologies including the number of patients and images per patient that were used to generate these datasets and to evaluate the performance of the proposed approach to differentiate between different laryngeal histopathologies:

- *Dataset II* CE + NBI images of the benign histopathologies. 20 patients with 890 images labeled into four groups: cyst, polyp & reinke’s edema, papilloma, and dysplasia mild.
- *Dataset III* 465 CE + NBI images belonging to 11 patients diagnosed with malignant histopathologies labeled into three groups: dysplasia severe, carcinoma in situ and carcinoma.
- *Dataset IV* CE + NBI images belonging to 31 patients with benign and malignant histopathologies that included a total of 1355 images labeled into two groups: benign and malignant.

### Qualitative analysis

**Fig. 6:**
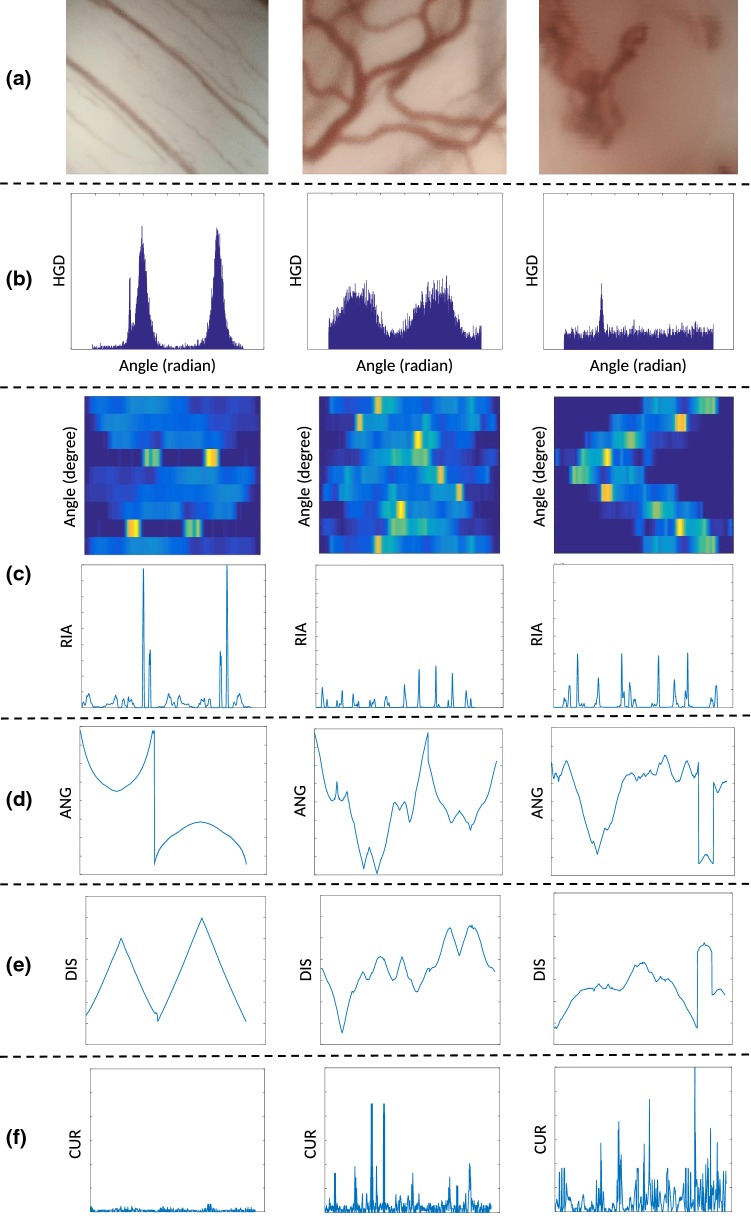
Five indicators for three different vascular patterns in CE + NBI images: **a** original image, **b** HGD indicator, **c** RIA indicator, **d** ANG indicator, **e** DIS indicator, **f** CUR indicator

Figure 6 shows the five indicators for three different vascular patterns. The indicators have qualitative characteristics that can be used to differentiate between different vascular patterns. A visual analysis of the indicators allows the following observation:

- The HGD indicator shows changes in the energy concentration with respect to the angle of the gradient vectors. Parallel vessel patterns show energy concentration of the gradient vector in two angles, while more chaotic vessel structures show a leakage in the energy distribution and even an equal distribution of energies (flat indicator) in the presence of spiral vessel patterns. It is possible to assume that the energy and energy-related characteristics of the HGD indicator can differentiate between different vascular patterns.
- The matrix of row averages for each rotation angle of the RIA indicator displays highly concentrated energies in two rotational angles when vessel patterns are parallel. This produces a final RIA containing a few number of main peaks of high amplitude. The more the vessel patterns become chaotic, the quantity of peaks and the energy leakage increase in the RIA. Energy-related features can therefore be used for characterizing vessel patterns.
- The displayed signal for both ANG and DIS indicators (Fig. 6) are a concatenated version for several vessel segments. This is why some signal discontinuities can be observed in the indicators. Disrespecting these discontinuities, we can observe that a vessel with significant curve patterns produces ANG and DIS indicators involving an increased number of changes per distance unit. This means that the quantity of changes of sign in their derivatives and the polynomial fitting errors will be higher for disorder patterns than for ordered ones, making it more suitable for distinguishing between patterns.
- CUR indicator variance increases when the vessel patterns become disorder. This is mainly because disorder patterns involve a higher number of loops and therefore more significant curvature’s values. For this indicator the features are also based on energies and peaks in the signal and also statistical values as variance.

Following this analysis, 24 features extracted from the 5 indicators are proposed for assessing vessel patterns, explained in the following.

*HGD features* Four features are proposed from the HGD indicator. The first, second, and third features, $F_1$, $F_2$, and $F_3$, are simply computed as the total energy and the minimal value of the HGD indicator, and as the difference between the maximum and minimum value of the indicator, respectively.

4 $$\begin{aligned} F_1&= \sum \limits _{g=1}^{N_\mathrm{HGD}} \hbox {HGD}^2(g) \end{aligned}$$

5 $$\begin{aligned} F_2&= \min _g[\hbox {HGD}(g)] \end{aligned}$$

6 $$\begin{aligned} F_3&= \max _g[\hbox {HGD}(g)] - \min _k[\hbox {HGD}(g)] \end{aligned}$$

where *g* correspond to the sample index of the indicator and $N_\mathrm{HGD}$ correspond to the total number of samples of the HGD indicator. The fourth HGD indicator $F_4$ intends to assess localized energy concentration of the indicator’s peaks. For that, significant peaks in HGD are first identified using a simple signal peak detector. Let $\hbox {on}_i$ and $\hbox {off}_i$ being the onset and offset of the HGD peak waveform *i* ($\hbox {HGDP}_i$) and $n_p$ the number of significant peaks identified in the HGD indicator. Then $F_4$ is computed as the ratio between the sum of the energy of the peaks of HGD and its total energy.

7 $$\begin{aligned} F_4 = \frac{\sum \limits _{1}^{n_p} \left[ \sum \limits _{\mathrm{on}_i}^{\mathrm{off}_i} \hbox {HGDP}^2_i\right] }{F_1} \end{aligned}$$

*RIA features* Four features are extracted from the RIA indicator. The first two features, $F_5$ and $F_6$, are computed as the total energy and as the number of significant peaks in the RIA indicator, respectively.

8 $$\begin{aligned} F_5&= \sum \limits _{g=1}^{N_\mathrm{RIA}} \hbox {RIA}^2(g) \end{aligned}$$

9 $$\begin{aligned} F_6&= \hbox {Peaks[RIA]} \end{aligned}$$

where *g* correspond to the sample index of the indicator, $N_\mathrm{RIA}$ correspond to the total number of samples of the RIA indicator and *Peaks* denote a function for significant signal peaks detection using a standard peak detector. For the third RIA feature $F_7$, a similar approach than for the computation of $F_4$ is proposed but using the average of the peak energies instead of the summation.

10 $$\begin{aligned} F_7 = \frac{\frac{1}{n_p}\sum \limits _{1}^{n_p} \left[ \sum \limits _{\mathrm{on}_i}^{\mathrm{off}_i} \hbox {RIA}^2_i\right] }{F_5} \end{aligned}$$

The fourth RIA feature $F_8$ is computed as the average of the ratios between amplitude and width of each peak waveform of the indicator.

11 $$\begin{aligned} F_8 = \frac{1}{n_p}\sum \limits _{1}^{n_p} \left[ \frac{\hbox {Amplitude}\, (\hbox {RIAP}_i)}{\hbox {Width}\,(\hbox {RIAP}_i)}\right] \end{aligned}$$

where $\hbox {RIAP}_i$ correspond to the RIA peak waveform *i*, $n_p$ to the number of identified RIA peaks and amplitude and width correspond to functions that compute the amplitude and width of each peak waveform.

*ANG features* Six features are extracted from the ANG indicator. One of the main characteristic of the ANG and DIS indicators is the change of sign in the derivative. Therefore, the first four ANG features, $F_9$, $F_{10}$, $F_{11}$ and $F_{12}$, are computed by exploiting this characteristic. Let *M* be the number of vessel segments identified in an image. For each vessel segment *m*, the derivative of the ANG indicator is first computed using the derivative filter presented in [22]. Then, the number of changes of sign $s_m$ is computed for each segment *m* and is used for computing the features. $F_9$, $F_{10}$, $F_{11}$ and $F_{12}$ are computed as the mean of $s_m$, the total number of changes of sign in an image, the maximal and the median of $s_m$, respectively.

12 $$\begin{aligned} F_9&= \frac{1}{M} \sum \limits _{m=1}^{M}\,s_m \end{aligned}$$

13 $$\begin{aligned} F_{10}&= \sum \limits _{m=1}^{M}\,s_m \end{aligned}$$

14 $$\begin{aligned} F_{11}&= \max _m\,[s_m] \end{aligned}$$

15 $$\begin{aligned} F_{12}&= \hbox {median}\,[s_m] \end{aligned}$$

Additionally, two features are computed based on the error $e_m$ of a 3rd degree polynomial fitting for each vessel segment *m*.

16 $$\begin{aligned} F_{13}&= \frac{1}{M} \sum \limits _{m=1}^{M}\,e_m \end{aligned}$$

17 $$\begin{aligned} F_{14}&= \hbox {median}\,[e_m] \end{aligned}$$

*DIS features* Six features were extracted from the DIS indicator ($F_{15}$ to $F_{20}$) using the same equations which were explained for the ANG indicator.

*CUR features* Four features are proposed from the CUR indicator. The first three CUR features, $F_{21}$, $F_{22}$ and $F_{23}$ are simply computed as the total energy, the number of significant peaks and the variance of the CUR indicator.

18 $$\begin{aligned} F_{21}&= \sum \limits _{g=1}^{N_\mathrm{CUR}} \hbox {CUR}^2(g) \end{aligned}$$

19 $$\begin{aligned} F_{22}&= \hbox {Variance [CUR]} \end{aligned}$$

20 $$\begin{aligned} F_{23}&= \hbox {Peaks [CUR]} \end{aligned}$$

where *g* correspond to the sample index of the indicator and $N_\mathrm{CUR}$ correspond to the total number of samples of the CUR indicator. The fourth CUR feature $F_{24}$ takes into account the observation that more chaotic vessel patterns will result in a bigger number of curves whose curvature level also is bigger. This is why we proposed as feature the number of signal peaks times the amplitude of the peak.

21 $$\begin{aligned} F_{24} = n_p \times \sum \limits _{1}^{n_p} \hbox {Amplitude (CUR)} \end{aligned}$$

The approach was implemented in MATLAB R2016b and executed on a PC with a CPU operating at 2.30 GHz resulting in an execution time of 4.02 seconds per image for image preprocessing, indicator computation, and feature extraction.

### Features classification performances

SVM, k-nearest neighbors (kNN), and random forests (RF) were used to classify CE + NBI images first based on three different vascular patterns (database I) and then based on the different histopathologies of the larynx (database II, III, and IV).

SVM performs classification by finding the hyperplane that maximizes the margin between the classes. The objective of the SVM algorithm is to find an optimal hyperplane in an N-dimensional space that distinctly classifies the data points. In this study, SVM with polykernel and radial basis function (RBF) kernel were used in order to classify linear and nonlinear separable data, respectively. The grid search method was used in order to optimize the SVM parameters using tenfold cross-validation and the classification was performed using a sequential minimal optimization (SMO) algorithm. In SVM Polykernel, there is one important parameter to optimize which is *C*, while in SVM with RBF kernel, there are two main parameters to optimize which are *C* and $\gamma$. *C* is the regulation parameter that controls the cost of misclassification on the training data and $\gamma$ is the kernel parameter that defines how far the influence of a single training example reaches. In our study, we decided to make the range of *C* and $\gamma$ from 0.01 to 1000 with 10 times increment. The SVM with Polykernel performed the best with $C = 1$ and SVM with RBF kernel showed the best performance with $C = 1$ and $\gamma = 0.01$. Furthermore, for solving the multi-class problem, a pairwise classifier trained the SVM to assign features into multi-class [23–25].

kNN is a nonparametric method and performs classification by finding the most similar data points in the training data and making an educated guess based on their classifications. The input consists of the *k* closest training examples in the feature space and the output is a class membership. In this study and in order to classify and assign a new sample to a new class, the distance of a sample was calculated using Euclidean distance algorithm. In kNN, *k* is the main parameter to optimize. For that, we used grid search method to find the optimized value with a range of *k* from 1 to 10 with step size equal to 1 and used tenfold cross-validation to select the best value. The classifier showed the best performance with $k = 3$ [26].

RF is an ensemble learning method for classification that operates by constructing a multitude of decision trees at training time and outputting the class. In this study, RF was trained via the bagging method. Bagging consists of randomly sampling subsets of the training data, fitting a model to these smaller data sets, and aggregating the predictions. Hence, instead of searching greedily for the best predictors to create branches, it randomly samples elements of the predictor space, thus adding more diversity and reducing the variance of the trees at the cost of equal or higher bias. There are many parameters in RF that can be optimized. In this study, we optimized only two important parameters which were the depth of the trees and number of estimators. The depth of the trees specifies the maximum depth of each tree and the number of estimators specifies the number of trees in the forest of the model. We made the range for the depth of the trees from 1 to 10 with step size equal to 1 and for number of estimators from 10 to 100 with step size equal to 5. The optimum parameters that were obtained after using grid search method with tenfold cross-validation were the depth of 8 with 50 trees [27].

A 24-dimensional space was fed into each classifier. The selected classifiers were applied by employing WEKA 3.8.1 as a machine learning tool. For all classification scenarios, a tenfold cross-validation was used for testing as well as for hyperparameter tuning. In order to measure the performance of each classifier, a confusion matrix was computed for each classification scenario and the accuracy, sensitivity, specificity, and area under the curve (AUC) receiver operating characteristics (ROC) were obtained from it. Tables 2, 3, 4, and 5 illustrate the classification results for each classifier. As we used tenfold cross-validation for all the classification scenarios, the values presented in these tables are the average results.

**Table 2:** Classification results using Polykernel SVM classifier

Database	Accuracy	Sensitivity	Specificity	AUC
Dataset I	0.973	0.980	0.983	0.977
Dataset II	0.846	0.819	0.942	0.917
Dataset III	0.864	0.856	0.931	0.917
Dataset IV	0.847	0.806	0.868	0.837

**Table 3:** Classification results using RBF SVM classifier

Database	Accuracy	Sensitivity	Specificity	AUC
Dataset I	0.968	0.976	0.978	0.973
Dataset II	0.816	0.757	0.926	0.901
Dataset III	0.873	0.864	0.931	0.921
Dataset IV	0.837	0.834	0.839	0.837

**Table 4:** Classification results using kNN classifier

Database	Accuracy	Sensitivity	Specificity	AUC
Dataset I	0.965	0.974	0.978	0.989
Dataset II	0.892	0.879	0.958	0.969
Dataset III	0.877	0.873	0.939	0.956
Dataset IV	0.912	0.871	0.933	0.953

**Table 5:** Classification results using RF classifier

Database	Accuracy	Sensitivity	Specificity	AUC
Dataset I	0.966	0.975	0.979	0.996
Dataset II	0.906	0.900	0.965	0.981
Dataset III	0.884	0.879	0.943	0.973
Dataset IV	0.911	0.939	0.858	0.979

## Discussion

To our knowledge, this is the first study on automatic characterization of vascular patterns in CE + NBI images with classification of the images using a set of features describing the level of disorder of vascular patterns.

Regarding the evaluation of the vascular structure in the CE images, there is a study [13] which classified the vascular patterns in ECE images into five groups. This classification was matched to the final diagnosis, with accuracy in the differential diagnosis between normal tissue and hyperplasia versus mild dysplasia and carcinoma of 97.6%. The result of this classification was based on the experience of the clinicians with a risk of subjective interpretation of vascular patterns in CE images [13, 16]. In contrast to that, we used an automated algorithm to characterize the level of disorder of vascular patterns. This method showed the ability to differentiate between three different vascular patterns with the accuracy, sensitivity, specificity, and AUC of over 96%. For the final diagnosis based on the vascular patterns in the ECE images of the vocal fold, [13, 15] reported the difficulty in differentiation between hyperplasia and low to moderate dysplasia. In comparison with our study, a different classification was used for laryngeal histopathologies and the RF classifier showed the best results to differentiate between benign histopathologies with an accuracy of $90\%$, a sensitivity of $90\%$, a specificity of $96\%$, and AUC of 98%.

For the evaluation of the cellular architecture of the most superficial mucosa in the head and neck area, according to [28] CE has an accuracy of 72–92%, a sensitivity of 77–100%, and a specificity of 66–100% to diagnose benign and malignant head and neck mucosal lesions. These results depend on the experience of clinicians and are based on the evaluation of cellular structures. Our proposed approach can perform an automatic differentiation between benign and malignant lesions based on vascular structure. All classifiers resulted in accuracy, sensitivity, specificity, and AUC of over $83\%$. The studies that focus on the cellular structure of the laryngeal tissue in CE reported the difficulty in diagnostic differentiation of carcinoma in situ from carcinoma [2], as well as dysplasia severe from carcinoma in situ and carcinoma [10, 29]. Our approach has the potential to solve these problems by distinguishing between three malignant histopathologies. For that, RF classifier showed the best results with the accuracy, sensitivity, specificity, and AUC of 88%, 87%, 94% and $97\%$, respectively.

## Conclusion

Based on the results, the presented approach could provide a confident way for clinicians to interpret vascular patterns in CE + NBI images with high accuracy. It also confirms the relevance of the vascular structures to the laryngeal histopathologies and to the stage of laryngeal cancer. Our approach has the potential to operate as an assisting system to help the clinicians make the final decision about the histopathology of the laryngeal tissue in the routine and surgical procedures.

As a first work in this field, our main objective was to propose an approach for characterization of the vascular patterns. Based on the discussion with clinicians and their requirements, we planned first to test the ability of the algorithm to differentiate between benign and malignant cases and then test the performance of the algorithm in each group (benign and malignant) to differentiate between different histopathologies. The next step of our work will be a multi-class classification with other features and considering all benign and malignant histopathologies. Further work is necessary to improve the results by computing more indicators, applying other feature extraction methods and implementing feature selection techniques to evaluate the influence of each class of features on the final results.

Also, the presented CE problems seem to be an ideal basis for machine learning approaches such as shown by [5, 6] using texture-based features and CNNs. This is possible when a high amount of data is available. CE is not a routine procedure in the clinical settings which caused a limitation on the number of patients available for our study. This problem also led to other limitations in the variety of histopathologies, especially in the benign cases. Therefore, the classification scenario of the benign cases was conducted with only available histopathologies. Hence, increasing the number of images per each dataset, testing the algorithm with other available classification of vascular patterns in CE images, and applying other classification methods are suggested for the future.
